# Recent-onset atrial fibrillation: challenges and opportunities

**DOI:** 10.1093/eurheartj/ehaf478

**Published:** 2025-08-28

**Authors:** Emma Svennberg, Ben Freedman, Jason G Andrade, Matteo Anselmino, Yitschak Biton, Giuseppe Boriani, Axel Brandes, Claire M Buckley, Alan Cameron, J L Clua-Espuny, Harry J G M Crijns, Søren Zöga Diederichsen, Wolfram Doehner, Helena Dominguez, David Duncker, Laurent Fauchier, Taya Glotzer, Yutao (Sheila) Guo, Karl Georg Haeusler, Moti Haim, Jeff S Healey, Jeroen M Hendriks, Mellanie True Hills, Gerhard Hindricks, F D Richard Hobbs, Linda S Johnson, Boyoung Joung, Hooman Kamel, Paulus Kirchhof, Deirdre A Lane, Lars-Åke Levin, Gregory Y H Lip, Shaowen Liu, Trudie Lobban, Peter W Macfarlane, Georges H Mairesse, Gregory M Marcus, Peter A Noseworthy, George Ntaios, Jessica J Orchard, Rod Passman, Daniel D Reidpath, James A Reiffel, Antonio Luiz Ribeiro, Lena Rivard, Prashanthan Sanders, Roopinder K Sandhu, Renate B Schnabel, Konstantinos C Siontis, Luciano A Sposato, Stavros Stavrakis, Steven R Steinhubl, Jesper H Svendsen, Andrew W Teh, Sakis Themistoclakis, Robert G Tieleman, A John Camm

**Affiliations:** Department of Medicine, Karolinska Institutet, Karolinska University Hospital Huddinge, Stockholm SE-141 86, Sweden; Heart Research Institute, Charles Perkins Centre, Faculty of Medicine and Health, The University of Sydney, Sydney, Australia; Cardiology Department, Concord Hospital, The University of Sydney, Sydney, Australia; Department of Medicine, Vancouver General Hospital, Vancouver, BC, Canada; Division of Cardiology, Cardiovascular and Thoracic Department, Città della Salute e della Scienza di Torino Hospital, Turin, Italy; Department of Medical Sciences, University of Turin, Turin, Italy; Heart Institute, Hadassah Medical Center and Faculty of Medicine, Hebrew University of Jerusalem, Jerusalem 9112002, Israel; Cardiology Division, Department of Biomedical, Metabolic and Neural Sciences, University of Modena and Reggio Emilia, Policlinico di Modena, Modena, Italy; Department of Cardiology, Esbjerg Hospital, University Hospital of Southern Denmark, Esbjerg, Denmark; Department of Regional Health Research, University of Southern Denmark, Esbjerg, Denmark; Health Service Executive of Ireland and School of Public Health, University College Cork, Cork, Ireland; School of Cardiovascular and Metabolic Health, University of Glasgow, Glasgow, UK; Jordi Gol University Institute for Primary Care Research (IDIAP Jordi Gol), Catalonia, Spain; Catalan Health Institute (ICS), SAP Terres de L’Ebre, Primary Care Health Tortosa-est, Tortosa 43500, Spain; Maastricht University Medical Centre (MUMC) and Cardiovascular Research Institute (CARIM), Maastricht, The Netherlands; Department of Cardiology, The Heart Centre, Copenhagen University Hospital—Rigshospitalet, Copenhagen, Denmark; Berlin Institute of Health-Center for Regenerative Therapies, Berlin, Germany; Deutsches Herzzentrum der Charité, Campus Virchow Klinikum, Charité - Universitätsmedizin Berlin, Berlin, Germany; German Centre for Cardiovascular Research (DZHK), Partner Site Berlin, Berlin, Germany; Center for Stroke Research, Charité Universitätsmedizin Berlin, Berlin, Germany; Department of Cardiology, Bispebjerg and Frederiksberg Hospital, Copenhagen, Denmark; Department of Biomedicine, University of Copenhagen, Copenhagen, Denmark; Hannover Heart Rhythm Center, Department of Cardiology and Angiology, Hannover Medical School, Hannover, Germany; Department of Cardiology, Centre Hospitalier Universitaire Trousseau et Faculté de Médecine, Université François Rabelais, Tours, France; Hackensack Meridian School of Medicine, Hackensack University Medical Center, New Jersey, USA; Chinese PLA Medical School, Pulmonary Vessel and Thrombotic Disease, Chinese PLA General Hospital, Beijing, China; Department of Neurology, Universitätsklinikum Ulm, Ulm, Germany; Cardiology Department, Soroka University Medical Center, Ben-Gurion University of the Negev, Beer-Sheva, Israel; Population Health Research Institute, McMaster University, Hamilton, ON, Canada; Department of Nursing, Maastricht University Medical Center+, Maastricht, The Netherlands; Department of Health Services Research, Care and Public Health Research Institute, Maastricht University, Maastricht, The Netherlands; Centre for Heart Rhythm Disorders, University of Adelaide, Adelaide, Australia; StopAfib.org, American Foundation for Women’s Health, Decatur, TX, USA; Deutsches Herzzentrum der Charité, Department of Cardiology, Angiology and Intensive Care Medicine, Charitéplatz 1, Berlin 10117, Germany; Oxford Institute of Digital Health, Oxford Primary Care, University of Oxford, Oxford, UK; Department of Clinical Sciences, Lund University, Malmö, Sweden; Division of Cardiology, Severance Hospital, Yonsei University College of Medicine, Seoul, South Korea; Clinical and Translational Neuroscience Unit, Feil Family Brain and Mind Research Institute and Department of Neurology, Weill Cornell Medicine, New York, NY, USA; Department of Cardiology, University Heart and Vascular Center Hamburg, University Hospital Hamburg Eppendorf, Hamburg, Germany; German Center for Cardiovascular Research (DZHK), Partner Site Hamburg-Kiel-Lübeck, Hamburg, Germany; Institute of Cardiovascular Sciences, University of Birmingham, Birmingham, UK; Department of Cardiovascular and Metabolic Medicine, University of Liverpool, Liverpool, UK; Liverpool Centre for Cardiovascular Sciences, University of Liverpool, Liverpool John Moores University, and Liverpool Heart and Chest Hospital, Liverpool, UK; Department of Clinical Medicine, Danish Center for Health Services Research, Aalborg University, Aalborg, Denmark; Department of Health, Medicine and Caring Sciences, Linköping University, Linköping SE-581 83, Sweden; Liverpool Centre for Cardiovascular Sciences, University of Liverpool, Liverpool John Moores University, and Liverpool Heart and Chest Hospital, Liverpool, UK; Department of Clinical Medicine, Danish Center for Health Services Research, Aalborg University, Aalborg, Denmark; Department of Cardiology Shanghai General Hospital, Shanghai Jiao Tong University School of Medicine, Shanghai, China; Arrhythmia-Alliance (A-A) and Atrial Fibrillation Association (AF Assoc), Celixir House, Stratford Business & Technology Park, Innovation Way, Stratford-upon-Avon, Warwickshire, UK; School of Health and Wellbeing Electrocardiology Section Level 1, New Lister Building Royal Infirmary Glasgow, Glasgow, UK; Cardiologie Electrophysiologie, Cliniques du Sud-Luxembourg, Rue des déportés 137, Arlon B 6700, Belgium; Division of Cardiology, University of California, San Francisco, California, USA; Department of Cardiovascular Medicine, Mayo Clinic, Rochester, MN, USA; 1st Propaedeutic Department of Internal Medicine, AHEPA University Hospital, Aristotle University of Thessaloniki, Thessaloniki 54636, Greece; Sydney School of Public Health, The University of Sydney, Sydney, Australia; Department of Medicine, Northwestern University Feinberg School of Medicine, Northwestern University Chicago, Chicago, Illinois, USA; Institute for Global Health and Development, Queen Margaret University, Dunfermline, UK; Columbia University Vagelos College of Physicians and Surgeons, New York City, NY, USA; Department of Internal Medicine, Faculdade de Medicina, and Telehealth Center and Cardiology Service, Hospital das Clínicas, Universidade Federal de Minas Gerais, Belo Horizonte, Brazil; Department of Cardiology, Montreal Heart Institute, Université de Montréal, Montréal, Canada; Centre for Heart Rhythm Disorders, University of Adelaide, Adelaide, Australia; Department of Cardiac Sciences, Libin Cardiovascular Institute, University of Calgary, Calgary, AB, Canada; Department of Cardiology, University Heart and Vascular Center Hamburg, University Medical Center Hamburg-Eppendorf, Hamburg, Germany; Department of Cardiovascular Medicine, Mayo Clinic, Rochester, MN, USA; Department of Clinical Neurological Sciences, Western University, London, ON, Canada; Department of Medicine, Cardiovascular Section, Health Sciences Center, Cardiovascular Section University of Oklahoma, Oklahoma City, USA; Weldon School of Biomedical Engineering, Purdue University, West Lafayette, IN, USA; Department of Cardiology, The Heart Centre, Copenhagen University Hospital—Rigshospitalet, Copenhagen, Denmark; Department of Clinical Medicine, University of Copenhagen, Copenhagen, Denmark; Department of Cardiology, Eastern Health Clinical School, Box Hill Hospital, Monash University, Victoria, Australia; Department of Cardiology, Austin Hospital Clinical School, The University of Melbourne, Victoria, Australia; Dell’Angelo Hospital, Venice, Mestre, Italy; Department of Cardiology, Martini Hospital Groningen, Groningen, The Netherlands; Department of Cardiology, University Medical Center Groningen, University of Groningen, Groningen, The Netherlands; City St. George's University of London, London, UK

**Keywords:** Atrial fibrillation, Screening, Device-detected atrial fibrillation, Subclinical atrial fibrillation

## Abstract

Atrial fibrillation (AF) is increasingly diagnosed early, close to its first occurrence due to: (i) increased public awareness with self-screening; (ii) health care initiatives including population screening and opportunistic case finding; and (iii) increased use and surveillance of implantable cardiac devices. At its onset, AF is often low burden, and cardiovascular co-morbidities may be absent or at an early stage. Thus, the management of recent-onset AF has become an issue of growing importance. Professional guidelines have traditionally focused on anticoagulant thromboprophylaxis, generally recommending a cautious approach to rhythm control, and priority has been given to rate control to alleviate symptoms. In recent guidelines, the importance of managing lifestyle and co-morbidities has increased. The AF-SCREEN collaboration proposes that a vigorous approach to active management of recent-onset AF may be warranted. This includes addressing co-morbidities and promoting healthy lifestyles to prevent the emergence or progression of AF and associated cardiovascular disease, as well as the initiation of active rhythm control ± anticoagulation to prevent AF-related morbidity and mortality, including stroke and heart failure (HF). Intuitively, intervention early after AF onset would be beneficial since lifestyle and co-morbidity management, plus rhythm control and anticoagulation, are important contributors to improved outcomes in patients with established AF, but robust evidence is lacking for recent-onset AF. There is a delicate balance between achieving favourable outcomes such as preventing strokes, HF and AF progression vs the complications and potential adverse effects of interventions. Given the serious long-term consequences, innovative approaches are necessary to determine the value and risks of initiating active therapy very early in the course of AF. More data are needed to guide the best management of recent-onset AF, bearing AF burden in mind. Long-term studies using large national databases linked to electronic medical records and rhythm monitoring devices offer excellent opportunities. Shorter-term studies focusing on reducing AF burden to slow AF progression and studies focusing on outcomes such as HF could be used in both randomized clinical trials and observational cohort studies.

## Graphical Abstract

**Graphical Abstract:**
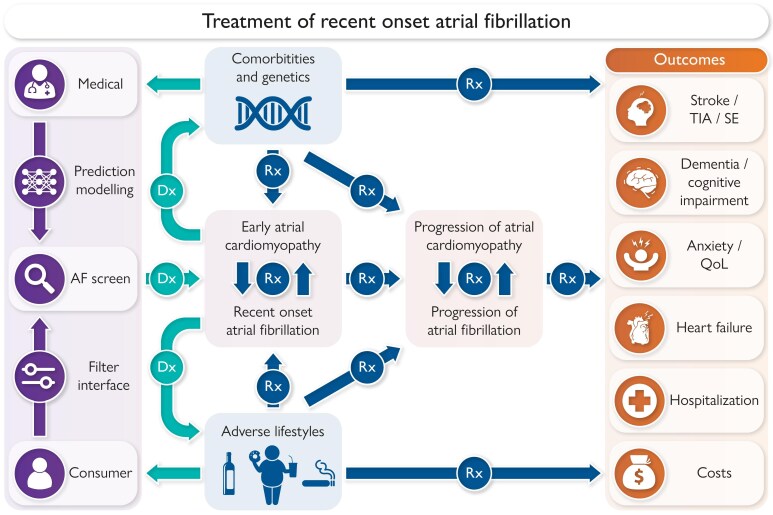
Diagnostic, therapeutic, and predictive pathways of recent-onset atrial fibrillation. The leftmost column (in purple) illustrates possible screening approaches. Diagnostic opportunities are indicated by arrows and marked with the abbreviation "Dx" (in green). The central boxes represent potential pathophysiological mechanisms underlying AF. Light blue boxes highlight identifiable causes of recent-onset AF. The rightmost column (in orange) outlines potential long-term outcomes associated with the condition. AF, Atrial fibrillation; Dx, diagnostic opportunities; Rx, treatment opportunity; TIA, transient ischaemic attack; SE, systemic embolism; QoL, quality of life.

## Introduction

Advances in atrial fibrillation (AF) management, including direct oral anticoagulants, improved detection technologies, and effective new rhythm control and thromboembolic reduction therapies, have sparked renewed interest in early diagnosis. The encouraging results of the EAST-AFNET 4 study, showing that early rhythm control, utilizing either anti-arrhythmic drugs or catheter ablation in experienced centres was superior to usual care, consisting of rate control for the majority, in reducing the composite outcome of death, stroke, and hospitalization for acute coronary syndrome or heart failure (HF) has led to an impetus to consider earlier intervention with rhythm control.^1^ Given that maintenance of sinus rhythm is easiest early in the disease process, these advances may be optimally harnessed when AF has recently developed.

The rise in use of consumer devices that monitor heart rhythm, and the increased use and surveillance of cardiac implanted electronic devices (CIEDs), have enabled AF to be detected early in the disease process, but even earlier detection could be facilitated through the prediction of AF. Artificial intelligence (AI), when applied to the electrocardiogram (ECG) or clinical risk factors, has shown accuracy in predicting AF before its clinical presentation.^2,3^ Developments in ECG monitoring and analysis, in conjunction with classical clinical risk characteristics and novel circulating biomarkers, imaging, and genetic profiling, promise earlier identification of patients at increased risk of developing AF. These developments enable early diagnosis and timely initiation of therapy, even in patients who have only rare and/or short episodes of arrhythmia.

Accordingly, a large group of patients is emerging for whom there is little management experience: patients who are at high risk of developing AF, recently had AF detected, or who have AF that has not yet become clinically apparent (‘pre-clinical’ AF) associated with atrial myopathy or underlying cardiovascular pathology which might not yet be irreversibly advanced. In the past, professional society guidelines generally recommended a restrained approach to rhythm control therapy, focusing mainly on symptom relief coupled with stroke thromboprophylaxis.^4,5^ Intuitively, it makes sense to deal with this arrhythmia effectively by ‘nipping-it-in-the-bud’ and by vigorously managing adverse lifestyles and co-morbidities.

Recent-onset AF is defined as AF at or within 1 year of its first presentation documented by an ECG, intracardiac electrogram, or wearable device, or with a history consistent with AF for no longer than 1 year (see Terminology).

For this review, the AF-SCREEN collaboration assembled current data to summarize evidence and knowledge gaps on recent-onset AF, condensing and refining consensus formulations as key points. Key points were voted on by the author group, and by members of the AF Screen International Collaboration. Key points included in the paper all achieved >90% agreement. These do not represent guidelines or formal recommendations but rather provide consensus views intended to provide a better understanding of the complexities and uncertainties of treatment in patients with recent-onset AF. Our report considers the diagnostic opportunities for uncovering recent-onset AF and the management pathways facilitated by an early diagnosis (*Graphical Abstract*).

## Terminology

In this document the term ‘clinical AF’ is used to denote AF diagnosed in a medical setting via a 12-lead ECG or on an ECG rhythm strip.

Device-detected AF (DDAF) without prior ECG diagnosis, also known as subclinical AF, is frequently reported in patients with CIEDs and requires inspection of intracardiac electrograms of atrial high-rate episodes.

Screening-detected AF is AF detected through a screening programme. In screening, photoplethysmography (PPG) might be used to suggest the presence of AF, but the diagnosis of screening-detected AF requires ECG confirmation.^6^

Atrial fibrillation which is triggered by a specific event such as surgery (post-operative AF) or a therapeutic/recreational drug is together described as ‘triggered or provoked AF’, and is commonly followed by recurrence of AF, often independent of the specific triggering event.

**Figure 1:**
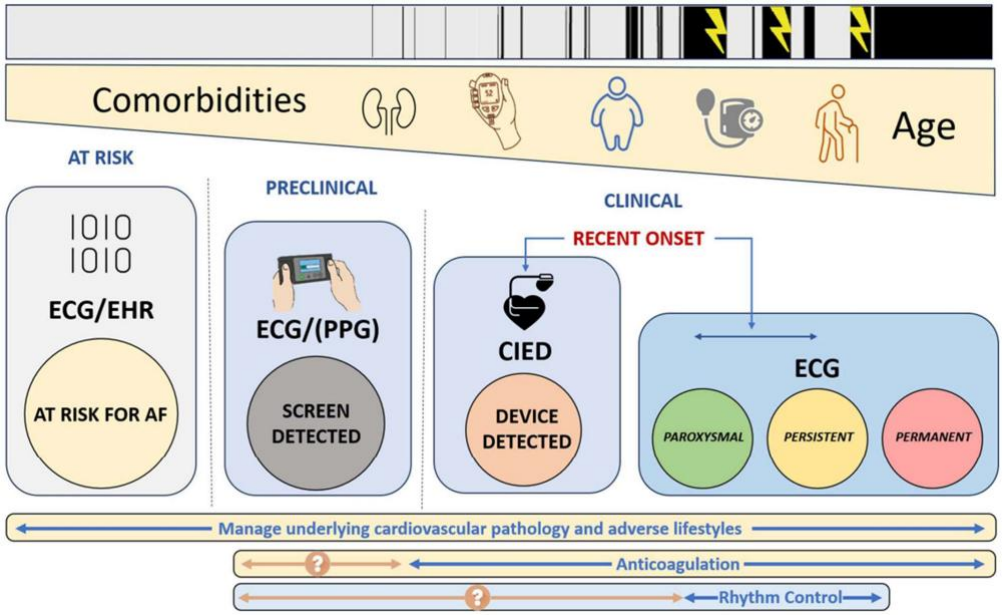
Figure showing the transition from at risk for developing atrial fibrillation, to the pre-clinical forms of atrial fibrillation, progression to recent-onset clinical atrial fibrillation and finally clinical atrial fibrillation and therapeutic strategies in place or available. ECG, electrocardiogram; EHR, electronical health care record; CIED, cardiac implantable electronic device; PPG, photoplethysmography

Several terms have been used to describe AF diagnosed soon after its initiation, including ‘early onset’, ‘new onset’, ‘first onset’ or ‘recent-onset’. There is no consensus on the time frame that should define this form of AF, which has ranged from 6 weeks [e.g. GARFIELD-AF—median .5 (.1–1.5) months]^7^ to 1 year [e.g. EAST-AFNET 4—median 36 (6.0–114.0) days]^1^ after its onset. In this review, we define ‘recent-onset’ as AF at or within 1 year of its first presentation documented by an ECG, intracardiac electrogram, or wearable ECG device, or with a history consistent with AF for no longer than 1 year. Recent-onset AF may be recognized by chance, for example, through an occupational or preoperative assessment or an AF screening programme. These patients tend to be younger and/or less symptomatic, have less co-morbid disease, and/or may engage in more adverse lifestyle behaviour than in patients where AF is diagnosed later in its course. There is little clinical experience with the management of this early form of AF, which may be low burden, and less likely to cause thromboembolism, and may not justify oral anticoagulation based on the conventional CHA_2_DS_2_-VASc scores derived from more traditional populations. Recent onset AF may be seen in younger populations, prone to nonadherence/non-persistence with therapy, and for them, a ‘pill-in-the-pocket’ anticoagulant management, when validated, might prove to be a better approach.^8^ Once recent-onset AF has been diagnosed, there is an opportunity to offer earlier treatment, including rhythm control and management of potential causative factors (atrial cardiomyopathy, underlying co-morbidities, and adverse lifestyles) to prevent possible adverse outcomes (*Figure 1*).

## Key points

**Table:** 

**1.**	Recent-onset AF can be defined as newly documented AF and a history consistent with AF for no longer than 1 year
**2.**	Recent-onset AF may be revealed by: medical or lay screening; risk stratification and subsequent monitoring; symptoms due to the arrhythmia; CIEDs; or from provocation by prescription or recreational drugs, acute disease, or interventions such as cardiac and non-cardiac surgery
**3.**	Recent-onset AF usually occurs in individuals with underlying cardiovascular disease, including atrial cardiomyopathy, or adverse lifestyles
**4.**	Recent-onset AF can progress from a predominantly trigger-dependent arrhythmia to a predominantly substrate-dependent arrhythmia
**5.**	Early rhythm control of recent-onset AF may be more effective than delayed treatment.
**6.**	Indications for early rhythm control in recent-onset AF may, but not necessarily include the presence of symptoms, the presence of atrial cardiomyopathy or underlying cardiovascular disease, the presence of HF, or any indication of AF progression
**7.**	Thromboprophylaxis with oral anticoagulants for patients with recent-onset AF may not be indicated in patients with device-detected paroxysmal AF episodes of short duration and a low estimated or measured AF burden, unless the patient has a high thromboembolic risk profile that would suggest a risk/benefit ratio in favour of anticoagulation
**8.**	Effective treatment of underlying cardiovascular conditions such as HF or hypertension and management of risk factors, including lifestyle modification, may prevent the onset of AF or reduce the likelihood of AF progression
**9.**	Early treatment of recent-onset AF may prevent deterioration of underlying co-morbid cardiovascular disease or reduce the likelihood of outcomes such as stroke or HF due to the arrhythmia itself

## Modes of detection of atrial fibrillation

Although irregular heart rhythms may be found on physical examination, devices using ECG (or PPG) are significantly more accurate than physical examination findings and are preferred for screening.^9,10^ According to ESC guidelines, PPG alone is not diagnostic for AF,^6^ whereas an ECG for 30 s [recorded on a medical grade device (including a watch or smart phone) regardless of number of leads] is sufficient for diagnosis if the rhythm strip is reviewed and the diagnosis confirmed.^9^ Longer monitoring times, e.g. enabled by devices and by consumer wearables, can detect AF with a low arrhythmia burden.

Consumer devices that enable self-screening are increasingly prevalent and utilize PPG and/or ECG,^11,12^ sometimes in combination, with incorporated automated algorithms for AF detection. Algorithms must balance sensitivity and specificity and exhibit good signal-to-noise ratios to maximize yield. While positive predictive values for AF detection are often reported as quite high, the underlying prevalence of AF among the populations studied may affect estimates of predictive value compared with that observed in the general population.^13–16^ Continued advances in algorithm accuracy with the use of AI hold the potential to refine the efficacy, feasibility, and cost-effectiveness of screening.

When first introduced, consumer-based screening using wearable devices such as smartwatches was limited by the fact that the younger population enrolled in the studies had the highest device (and self-screening) uptake but a low pre-test probability of AF detection, thus limiting the predictive value of a positive finding.^11,12,17^ Conversely, the most at-risk groups (older patients, lower socio-economic status groups) were less likely to self-screen with smart technology.^18,19^ However, older people are becoming more digitally savvy, and digitally savvy persons are becoming older.^20^ In patients with CIEDs, the prevalence of asymptomatic DDAF, lasting ≥5 min in patients with risk factors but no prior diagnosis of AF is ∼30%.^21^ Although most episodes resolve spontaneously, it is clear that DDAF is a marker of progression to clinical AF (6%–9%/patients per year in the NOAH-AFNET 6 and ARTESiA trials).^22–25^

## Prediction models of atrial fibrillation

Multiple AF prediction scores have been developed using prospectively screened cohorts, including the CHARGE AF,^26^ ARIC AF,^27^ Framingham Heart Study AF,^28^ HATCH score,^29^ C_2_HEST,^30^ and the HARMS2-AF,^31,32^ with varying simplicity and practicality. A systematic review of several of these scoring tools found moderate discriminative performance for predicting AF.

Newer models derived using machine learning have demonstrated strong discriminative performance but lack rigorous validation. These algorithms are developed from primary care electronic health records, have similar performance and limited prospective validation, but are more inclusive.^33,34^ Artificial intelligence-based AF prediction models using ECGs and combinations of clinical features and ECG data have been developed^2,3^ but their performance across different clinical settings is unknown, and they require fine-tuning when applied in different clinical scenarios.^35^

In theory, screening patients in the ‘pre-clinical’ phase of AF may provide an opportunity to address co-morbid conditions that increase the future risk of AF and underlying co-morbidities. However, to date, screening a high-risk population defined by an AF risk score has not been consistently demonstrated to reduce stroke, systemic embolism, or overall survival.^19,36–38^

Continued advancements in algorithmic precision, driven by collaborative efforts among researchers, healthcare providers, and technology companies, could transform AF diagnosis and facilitate proactive management of this prevalent cardiac condition to prevent outcomes other than stroke or death.

## Screening for atrial fibrillation in health care

Single time-point ECGs have successfully detected AF in non-randomized trials.^39^ However, as baseline detection via case-finding and wearables has increased, their utility for identifying non-paroxysmal AF has diminished, with recent randomized controlled trials (RCTs) yielding neutral results.^40–42^ Systematic screening with prolonged monitoring has improved detection, primarily of paroxysmal AF.^43^

Randomized controlled trials have investigated whether screening and subsequent OAC initiation reduce adverse outcomes.^19,37,38^ Despite high OAC uptake, only one trial showed a modest (4%) reduction in a composite endpoint (death, stroke, thromboembolism, major bleeding), while most remained neutral.^19^ Meta-analyses have reported a small but significant stroke reduction with screening.^44,45^

Large ongoing trials, including SAFER trial,^46^ Heartline (NCT04276441), and NOR-SCREEN trial (NCT05914883), aim to clarify optimal settings and strategies for screening and OAC treatment to improve outcomes like stroke.

## Consumer-based screening for atrial fibrillation

**Figure 2:**
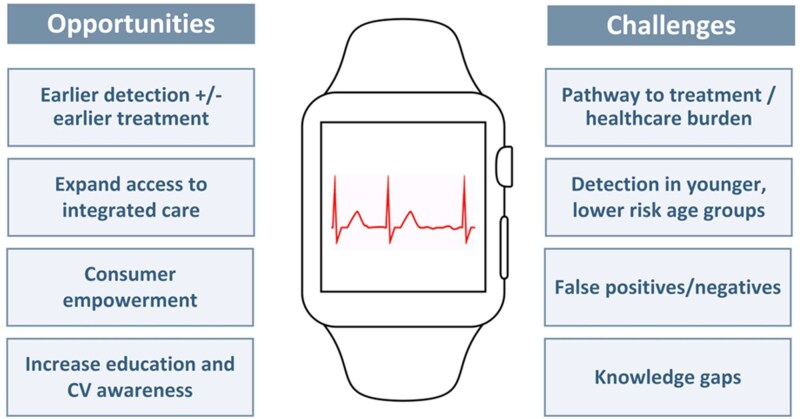
Opportunities and challenges from consumer-based screening for recent-onset atrial fibrillation

Consumer-based screening offers several key opportunities (*Figure 2*).^9^ Digital devices provide a platform for patient education about AF and an opportunity for consumers to examine and modify risk factors. These opportunities necessitate increasing the availability of integrated care that supports consumer-based screening and is not fixated solely on short-term stroke reduction.^47^

There are significant challenges in consumer-based screening, including determining the optimal pathway to reach a confirmatory diagnosis when an irregular rhythm is detected on PPG and determining optimal treatment pathways. An increase in self-detection results in more individuals seeking medical attention. Moreover, the potential for false negatives or positives from consumer-based screening may result in false reassurance or unwarranted anxiety for users and misallocation of healthcare resources.^48^ While consumer-based devices face challenges in the accurate classification of AF vs sinus rhythm, it is important to note that similar diagnostic uncertainty exists in clinical practice. Studies have shown that even trained healthcare providers, including general practitioners and cardiologists, may misinterpret ECGs or rhythm strips, particularly when AF is paroxysmal or when recordings are of suboptimal quality.^49,50^

## Early atrial cardiomyopathy

Atrial cardiomyopathy has been defined as ‘any complex of structural, architectural, contractile or electrophysiological changes affecting the atria with the potential to produce clinically relevant manifestations’.^51^ These changes are promoted by numerous risk factors, including genetic predisposition and age.^52,53^ Clinically relevant outcomes, including thromboembolism, HF, and mortality, are more likely to occur in the presence of atrial cardiomyopathy.

Biopsy studies in selected populations have reported pathological atrial changes such as vacuolar degeneration, inflammatory infiltrates, and fibrosis.^54^ Similar changes are seen in left ventricular cardiomyopathy, supporting the concept that the left atrium is also susceptible to cardiomyopathic changes.^54^ These pathological changes correlate with non-invasive markers of atrial cardiomyopathy, such as increased P-wave terminal force, N-terminal pro-B-type natriuretic peptide (NT-pro-BNP), or left atrial diameter.^55–57^

Experimental data and observational studies suggest that atrial cardiomyopathy promotes initiation, maintenance, and progression of AF. Atrial fibrillation, conversely, is a major accelerator of atrial cardiomyopathy.^58^ While ectopic activity appears to be required to trigger AF, structural features of the left atrium such as fibrosis may cause a re-entry prone substrate that allows for easier initiation and maintenance of AF.^59^ Subtle evidence of left atrial dysfunction is associated with future development of clinically apparent AF,^60^ supporting the concept that early atrial cardiomyopathic changes precede the development of the arrhythmia.

Whether screening for AF in patients with atrial cardiomyopathy is an effective way to detect recent-onset AF remains unknown. The potential of AF to worsen the underlying atrial cardiomyopathy may explain the beneficial effects of restoration of sinus rhythm, particularly early in the disease course.

**Figure 3:**
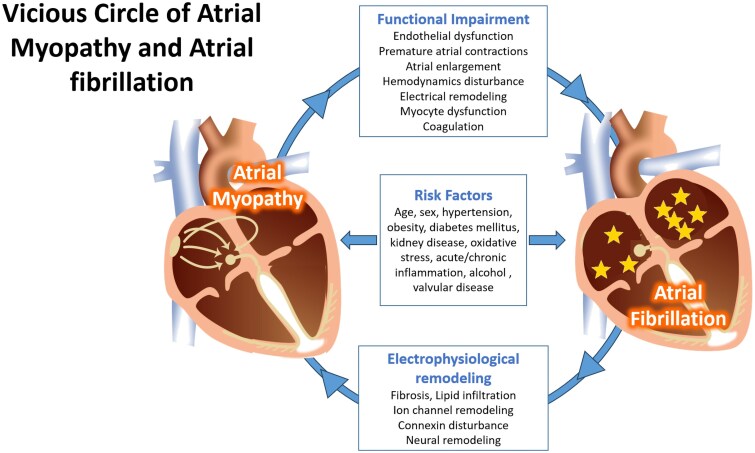
Vicious circle of atrial myopathy and atrial fibrillation

In addition to promoting the development and progression of AF, atrial cardiomyopathy also plays a role in some of the clinical sequelae of AF. This link has been most thoroughly investigated regarding the thromboembolic risk associated with AF.^61^ Among patients with AF, the extent of left atrial fibrosis as measured by cardiac magnetic resonance imaging, is strongly associated with the risk of stroke.^62,63^ The addition of blood biomarkers of myocyte injury and stretch, such as troponin and brain natriuretic peptide, as well as electrocardiographic markers of atrial remodelling, adds incremental value for stroke risk prediction in patients with AF^64–67^ (*Figure 3*).

However, atrial cardiomyopathy without ECG-documented AF did not justify anticoagulation after stroke in the ARCADIA trial.^61^ Nevertheless, separate from the issue of anticoagulation, early treatment of AF itself may reduce the progression of atrial cardiomyopathy and stroke risk.

## Progression of recent-onset atrial fibrillation

**Figure 4:**
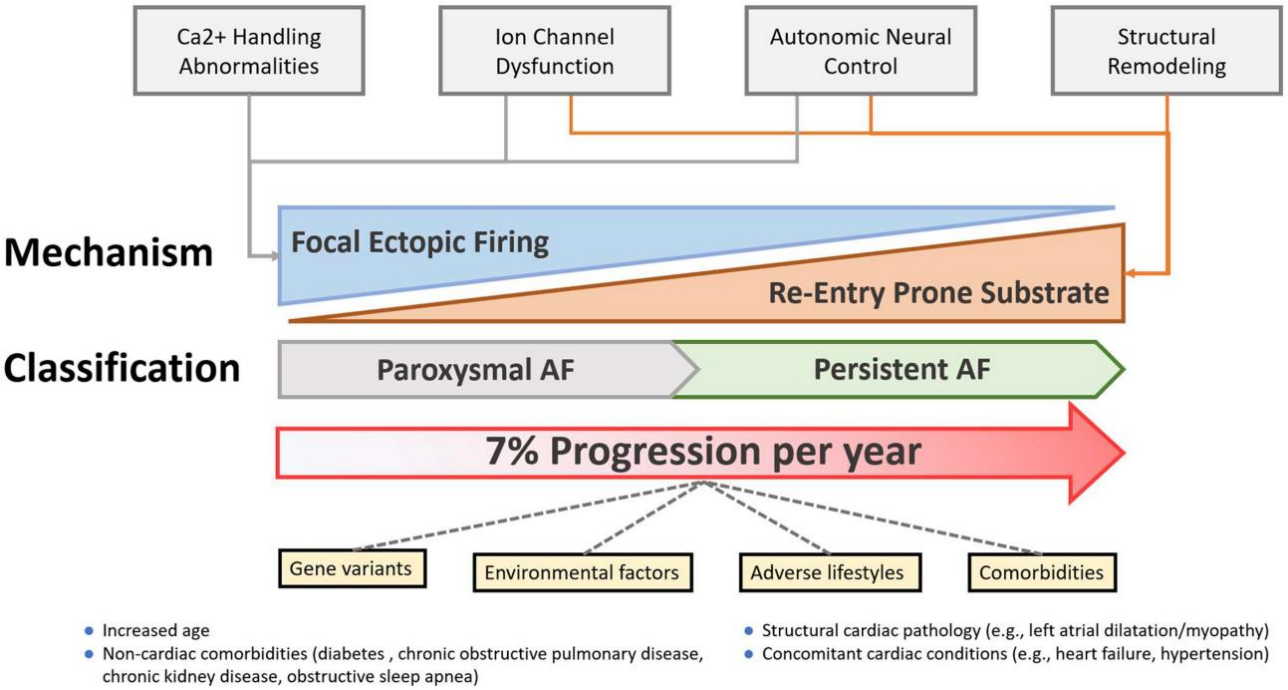
Mechanistic view of atrial fibrillation progression atrial fibrillation progresses from an electrical state with focal ectopic firing, to a substrate-based disease; factors that affect and perpetuate this change

Atrial fibrillation is a chronic progressive disease, characterized initially by arrhythmia exacerbations interspersed with quiescent periods. The rate of AF progression is slow and seems to depend on the presence of co-morbidities.^68^ During the initial paroxysmal phase, AF can manifest as an isolated electrical disorder, especially in young patients with repetitive pulmonary venous ectopic trigger activity that may perpetuate AF by electrical and structural remodelling over time. A gradual shift from a disease driven by focal firing to an arrhythmogenic substrate is illustrated in *Figure 4*. Episodes of very short duration (<30 s) irregular tachycardias, termed micro-AF, may progress to clinical AF.^69,70^ Several observational studies have shown that premature atrial contractions, atrial tachycardias, left atrial enlargement, and elevated NT-pro-BNP are associated with incident AF. These factors and elevated activity of other cardiometabolic disease processes also predict recurrent AF on rhythm control therapy and are associated with outcomes,^71^ highlighting potential similarities between factors associated with recent-onset AF, AF progression, and AF recurrence. These atrial myopathy markers are highly prevalent in most middle-aged populations, and studies of risk factors for progression to clinical AF in subjects with atrial myopathy are lacking.

Studies on the natural history of AF suggest that the rate of progression to non-paroxysmal AF may be higher during the first years following index diagnosis. In the Canadian Registry of AF, the rate of progression from paroxysmal to persistent AF was 8.6% at 1 year, but only reaching 36.3% at 10 years. However, depending on the co-morbidity profile, progression rates vary from as high as 25%–35% at 1 year, to 75% at 15 years of follow-up.^72^ For early stages of AF progression, rates may be lower. For instance, ECG-documented AF developed in 8.8%/year in the ASSERT and NOAH-AFNET 6 studies,^22,73^ which was slightly higher than the 6.5%/year in ARTESiA.^23^ This contrasts with the LOOP study where half of the patients with AF did not show AF progression, and one in four had complete remission of AF.^74^ Faster progression to ECG-documented AF has been observed in patients with DDAF lasting ≥24 h.^24,73^

Non-paroxysmal AF is associated with a higher risk of death, thromboembolism, and hospitalizations for HF.^75–77^ Events occur more frequently in patients experiencing AF progression, with peak adverse outcomes being observed during the ‘peri-progression period’.^78^ Consequently, there is growing interest in targeting the prevention of AF progression as a novel therapeutic endpoint.^77,79,80^ Studies such as RACE 3 and REVERSE-AF have demonstrated that lifestyle intervention is associated with delayed progression and regression of AF.^81,82^ In the RECORD-AF study and the ATHENA trial, anti-arrhythmic drug therapy was associated with lower rates of disease progression when compared with pharmacological rate-control or placebo, respectively.^83,84^ ATHENA also showed that anti-arrhythmic drug therapy with dronedarone reduced cardiovascular outcomes including stroke, in a *post hoc* analysis.^85^ Catheter ablation is more effective than anti-arrhythmic drug therapy for reducing arrhythmia burden, health care utilization, improving quality of life (QoL), and is also associated with lower rates of progression from paroxysmal to non-paroxysmal AF.^80,86,87^ Only a minority of patients (<3%) experience progression of AF in the first few years after catheter ablation.^79,80^ It is thought that the lower rate of progression is a result of catheter ablation being more effective in preventing recurrent AF than anti-arrhythmic drugs.

## Triggered atrial fibrillation

Recent-onset AF may occur spontaneously, or it may be provoked in a variety of settings. For example, adrenergic AF occurs during exercise, and vagotonic AF occurs when vagal nerve activity is intensified.^88^ Through many possible mechanisms a wide variety of therapeutic agents,^89^ such as anticancer drugs (e.g. anthracyclines, melphalan, cisplatin, and kinase inhibitors—ibrutinib),^90^ antipsychotics (e.g. clozapine and olanzapine) and bisphosphonates (e.g. zoledronic acid and alendronate) are associated with increased AF. Ivabradine is associated with the provocation of AF,^91^ but interestingly confers good rate control when specifically used to treat AF.^92^

Acute systemic illness (such as sepsis or pneumonia), the perioperative state (after cardiac or non-cardiac surgery), acute coronary syndrome, or acute HF may also stimulate the arrhythmia. The prognostic importance of such triggered (provoked or secondary) AF has recently gained increased attention.^93^ While traditionally considered a benign and transient arrhythmia, triggered AF is now recognized as a significant marker of adverse long-term prognosis, indicating a capacity to later develop sustained AF.^94^ Factors associated with systemic illness or perioperative state, such as stress, autonomic system dysregulation, electrolyte abnormalities, and neurohormonal changes, might act synergistically to unearth AF in patients with a predisposing AF substrate related to atrial myopathy and other co-morbidities.

Triggered AF may be a marker of vulnerability to AF, and if one trigger provokes the arrhythmia, others may do so too, and AF seems to become a recurrent problem in many of these patients. How to monitor such patients to identify recurrences is not yet well evaluated but the stroke risk is likely to be related to AF burden and the underlying cardiovascular profile. Therefore, the monitoring intensity should relate to the risk, and anticoagulation should be advised for those with a high risk of recurrence and high stroke risk scores. For example, recent data suggest that AF after non-cardiac surgery is associated with a stroke risk similar to that of clinical AF, although the post-operative AF was not well anticoagulated^95,96^ and OAC consideration should probably follow the same rules for triggered AF as it does for other AF scenarios.^6^ Ongoing randomized trials are anticipated to shed light on the effectiveness and safety of OAC for post-operative AF. Further, the optimal approach to post-hospitalization rhythm monitoring is unknown. Two-week monitoring with a patch ECG monitor has revealed a high rate of subsequent AF detection (approximately one in three patients within 1 year after the provoked new-onset AF episode).^97^

## Co-morbidities associated with emergence of atrial fibrillation

Atrial fibrillation is associated with underlying cardiovascular co-morbidities and adverse lifestyle habits, which predispose to AF and increase the risk of AF-related complications (*Table 1*).

**Table 1:** Comorbidities contributing to atrial fibrillation: underlying mechanisms, relevance to recent-onset AF, and treatment strategies

Co-morbidity	Prevalence of co-morbidity in clinical AF	Association with recent-onset AF	Potential mechanism	Treatment opportunities in recent-onset AF
Hypertension	49%–90%^98^	In screening studies increased AF detection was seen in hypertensive patients^99,100^	Hypertension leads to left ventricular hypertrophy, reduced compliance, increased stiffness, and elevated filling pressures, activating the sympathetic nervous and renin–angiotensin–aldosterone systems. These changes raise left atrial pressure, promoting fibrosis and conduction abnormalities that predispose to AF^98^	Treatment of hypertension, in particular with renin–angiotensin inhibitors could lead to early reverse remodelling and reduction of AF burden.^101^ Renal denervation for hypertension might prevent subclinical AF, possibly due to lowering blood pressure or direct autonomic effects on the heart^102^
Obesity	20%–40%^103^	For every unit increase in body mass index (BMI) there is a 4% increase in incident AF.^104^ Progression from paroxysmal to non-paroxysmal AF is associated with increasing BMI^105^	Obesity leads haemodynamic and structural changes, that in addition to cardiac adiposity, inflammation, fibrosis, oxidative stress, ion channel remodelling, and autonomic dysfunction might lead to AF.^106^ Obesity can predispose to or co-variate with other risk factors that might increase the risk of AF^107^	Weight loss reduce progression of AF.^81^ The GLP-agonist semaglutide reduces the risk of AF by 42% regardless of BMI in a meta-analysis of RCTs.^108^ Weight loss after initiation of Sodium–Glucose Cotransporter 2 (SGLT-2) inhibitor reduced the risk of AF
Heart failure	20%–30%^109^	A higher AF burden in patients with device-detected AF was associated with an increased risk of heart failure^110^	Structural remodelling, due to increased atrial pressure and overload leading to atrial dilatation,^111^ proinflammatory response,^112^ sympathetic nervous system activation,^113^ similar risk factor profiles and genetic factors^110^ can lead to the development of heart failure and AF	HF goal-directed quadruple HF therapy and cardiac resynchronization therapy reduces the risk of incident AF. Earlier HF treatment reduces the risks more than delayed therapy
Diabetes	15%–27%^114^	Excess risk of recent-onset AF in patients with poor glycaemic control^115,116^	Mechanisms for developing AF in diabetes include direct effects (so-called diabetic cardiomyopathy) as well as diabetes-associated HF, atherosclerotic vascular disease, and the prevalence of other co-morbidities^117^	Treatment with metformin and pioglitazone, may be associated with lower rates of new AF.^5,118^ Recent studies investigating SGLT2 inhibitors show a lower risk of incident AF and AF-related complications compared with untreated populations, although these studies were not specifically designed to detect AF^119,120^
OSA	21%–84%^121–123^	OSA increases new-onset AF and recurrent AF.^124^ There is also a possible ‘dose-response’ relationship between OSA severity and AF incidence, burden, and response to treatment^124^	Arrhythmogenesis in OSA is a multifactorial process characterized by a combination of acute atrial stimulation during hypopnoea on a background of chronic electrical, structural, and autonomic remodelling from chronic hypopnoea^124^	Optimal management of OSA may reduce AF incidence, AF progression, AF recurrences and symptoms.^125,126^ Treatment of OSA is focused on modifications during sleep, lifestyle modifications, institution of positive airway pressure, and implantable device therapy. In addition, no randomized trial has demonstrated that treatment of OSA can prevent AF or reduce the burden of AF,^127^ but this may be due to suboptimal durable treatments for OSA more than causal contributions of OSA to AF *per se*

Hence, several risk factors increase the risk of incident AF, and treatment of the risk factors might mitigate this risk.

## Adverse lifestyle factors

**Figure 5:**
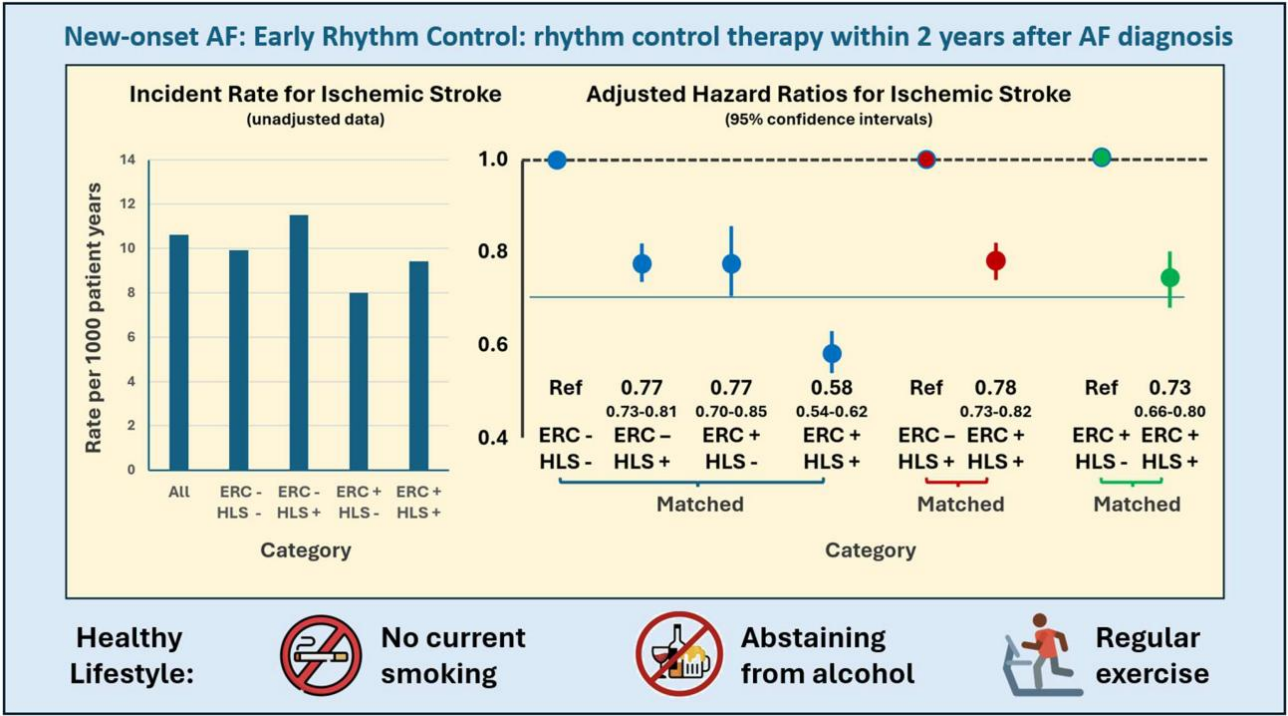
Outcomes associated with rhythm control stratified per lifestyle in recent-onset atrial fibrillation. Graph showing unadjusted and adjusted incidence of ischaemic stroke per presence or absence of early rhythm control ± a healthy lifestyle. ERC, early rhythm control; HLS, healthy lifestyle. Adapted from Lee *et al*.^128^

Adverse lifestyle factors increase the risk of AF, and using the HARMS2-AF score, individuals at risk of AF can be identified.^31^ Following the diagnosis of AF, modification of adverse lifestyle factors and treatment of underlying cardiovascular co-morbidities may augment the effectiveness of AF-specific therapies and confer additional benefits in reducing broader cardiovascular risk. Recent-onset AF may be an early signal that poor lifestyle or cardiovascular disease may be present and in need of attention (*Figure 5*).

### Insufficient physical activity

An inactive lifestyle or reduced cardiorespiratory fitness has been associated with a greater risk of AF, and routine physical activity contributes to a reduction in incident AF. Guideline-recommended levels of physical activity, engaging in at least 210 min per week, reduce incident AF.^129,130^

Recently, the ACTIVE-AF trial demonstrated that randomized assignment to more physical activity could reduce the burden of AF.^131^ These data suggest that in those with recent-onset AF, promotion of a physically active lifestyle should be encouraged.

### Alcohol consumption

Excessive drinkers face higher risks of first-time and recurrent AF. Recent analyses suggest that quitting alcohol may prevent AF.^132–134^

Alcohol causes immediate electrophysiologic effects, rendering the atria more prone to fibrillate.^135^ Per-protocol analyses of the I-STOP-AFib trial revealed alcohol to be the only modifiable trigger associated with an increased burden of AF.^136^ Objective data from wearable sensors have confirmed that just one drink of alcohol increases the risk of a discrete AF episode a few hours later.^133^

These data suggest that early detection of AF may identify individuals most likely to benefit from alcohol avoidance.

### Smoking and pulmonary disorders

Those who smoke tobacco experience a higher risk of AF,^137,138^ offspring of smoking parents are more prone to AF later in life,^139^ and second-hand smoke exposure increases AF risk.^140^ While the effects of smoking or smoking cessation on the burden of AF among those with the disease have not yet been directly investigated, recent-onset AF may reasonably provide a compelling incentive to motivate smoking cessation.

### Caffeine and recreational drugs

The most consumed recreational drug is caffeine. Contrary to conventional wisdom that caffeine promotes arrhythmias, the majority of studies have either failed to demonstrate a relationship with AF^141,142^ or suggest that coffee may protect against incident AF.^143,144^ In a randomized case-crossover trial, coffee did not influence the frequency of premature atrial contractions (known to be a potent predictor and trigger of AF).^145^

In contrast, individuals who use cannabis, cocaine, methamphetamine, and opiates exhibit a heightened risk of AF, suggesting that AF detected during screening might provide a reason to avoid recreational drugs.^146^ A recent analysis of UK Biobank participants failed to demonstrate a relationship between cannabis and incident AF, suggesting that less common recreational use may not incur a meaningful risk, but the finding might also be explained by the low-risk population studied.^147^

### Endurance athletes

Participation in endurance sports, such as cycling and cross-country skiing, performed over many years has been associated with a greater risk of developing AF. Previous studies indicated that this risk was mainly present in male athletes, but recent data indicate similar findings for female athletes.^148^ Traditionally, a reduction in physical activity has been recommended,^149^ but evidence is sparse, and it is currently under investigation in a RCT.^150^

## Treatment and outcomes

This section highlights evidence suggesting that early treatment, initiated soon after the detection of recent-onset AF, can significantly reduce the risk of adverse outcomes associated with the condition.

### Stroke

Clinical AF substantially increases ischaemic stroke risk, particularly in older patients with co-morbidities.^151^ While this risk is somewhat lower in paroxysmal vs persistent/permanent AF, anticoagulation is recommended across all AF patterns based on stroke risk.^152^ Stroke risk is also elevated in DDAF, even at low burden.^153,154^

Two RCTs have assessed OAC use in DDAF, often considered recent-onset. In NOAH-AFNET 6 (*n* = 2356), edoxaban (60 mg daily) was compared with aspirin or placebo.^22^ The trial was stopped early due to futility and safety concerns. Stroke/systemic embolism/cardiovascular death rates were similar between arms, but major bleeding and all-cause death were higher with edoxaban. Stroke incidence was lower than expected.^22^

In ARTESiA (*n* = 4012), patients with DDAF (6 min–24 h) and stroke risk factors were randomized to apixaban (5 mg b.i.d.) or aspirin.^23^ Stroke/systemic embolism occurred at .78% vs 1.24%/year [hazard ratio (HR) .63; 95% confidence interval (CI) .45–.88]. Apixaban reduced disabling/fatal strokes (HR, .51; 95% CI, .29–.88) but increased major bleeding (HR, 1.36 ITT; HR, 1.80 on-treatment), with no excess in fatal/intracranial bleeding.

A meta-analysis of both trials showed a 32% relative risk reduction in stroke/embolism and a 62% increase in major bleeding with OAC vs aspirin, without an increase in fatal bleeding or cardiovascular death.^153^ Given the absolute thromboembolic risk (∼1%/year), these findings suggest a modest net benefit of OAC.^153^

An ARTESiA substudy showed apixaban prevented 1.28 strokes/year and caused .68 major bleeds in patients with CHA_2_DS_2_-VASc >4. For those with scores ≤4, benefits and risks were closely balanced (.32 vs .28 events/year), underscoring the need for individualized decisions.^155^

Recognizing the limitations of sub-analyses, including small sample sizes, in a sub-analysis of NOAH-AFNET 4, the low rate of ischaemic stroke without anticoagulation extends to patients with long episodes of DDAF (≥24 h).^24^

Growing evidence suggests that rhythm control may reduce stroke risk by lowering AF burden.^156^ In the EAST-AFNET 4 trial, early rhythm control in recent-onset AF reduced stroke rates by one-third.^157^ Similar findings emerged from a *post hoc* analysis of the ATHENA trial with dronedarone.^85^ In EAST-AFNET 4, stroke reduction was mediated by maintenance of sinus rhythm,^158^ reinforcing the potential role of AF burden reduction. However, it remains uncertain whether early rhythm control alone can sufficiently reduce AF burden to eliminate the need for oral anticoagulation or other thromboprophylactic strategies.

### Cognitive impairment

Evidence linking established AF to cognitive impairment and dementia continues to grow.^159,160^ In women, AF may accelerate dementia progression.^161^ Proposed mechanisms include small vessel disease, infarcts, hypoperfusion, inflammation, and blood-brain barrier disruption.^162^ The impact of OAC, screening, ablation, cardioversion, and medication on cognition remains unclear.^163–165^ The AF burden threshold for cognitive effects is also unknown, with conflicting findings from continuous monitoring studies.^166,167^ In EAST-AFNET 4, early rhythm control showed no cognitive benefit via MoCA testing, though follow-up was short.^1^ In BRAIN-AF, patients with AF at low stroke risk (mean age 53, 26% women) randomized to rivaroxaban vs placebo had no significant difference in stroke, transient ischaemic attack, or cognitive decline after 3.7 years.^168^

As both AF and dementia are projected to rise, RCTs using standardized cognitive assessments are needed to determine whether interventions, including early AF treatment, can prevent cognitive decline.

### Heart failure/left ventricular dysfunction

In a systematic review and meta-analysis, the relative risk for the development of HF in AF patients, was estimated to be five times higher compared with patients without AF.^169^

Early rhythm control is considered effective and safe for patients with AF and HF. Studies, including sub-analyses of EAST-AFNET 4 and CABANA,^170,171^ and metanalyses, suggest that AF ablation is more effective in reducing adverse outcomes compared with anti-arrhythmic drug therapy.^172^ Pulmonary vein isolation has shown improved outcomes for patients with AF and heart failure with reduced ejection fraction (HFrEF), though its implementation may be limited by availability.^172–174^ Ongoing trials like CABA-HFPEF (NCT05508256) and EASThigh-AFNET 11 (NCT06324188) aim to determine if AF ablation benefits patients with HFpEF and AF or those with multiple co-morbidities.

Atrial fibrillation can also lead to annular dilation, causing functional mitral and tricuspid regurgitation. Atrial functional mitral regurgitation, resulting from annular-leaflet imbalance, was found in 6.5% of patients undergoing AF ablation.^175^ Patients maintaining continuous sinus rhythm post-ablation showed greater reductions in left atrial size and annular dimension and had lower rates of significant mitral regurgitation.

There is limited knowledge on whether early rhythm control can reduce the progression and burden of AF, potentially preventing or lessening the likelihood of HF.

### Hospitalization/emergency room visits

Atrial fibrillation-related hospitalizations are rising, placing a growing financial burden on healthcare systems.^176,177^ While rhythm control was linked to higher hospitalization rates in earlier rate vs rhythm-control trials,^178,179^ timely AF detection can enable early intervention and potentially reduce disease progression and hospital admissions. Early rhythm control has been associated with fewer cardiovascular events and lower rates of unplanned hospitalizations for HF or acute coronary syndrome.^1,180^

In MAFA II, mobile app–supported integrated early AF management that lowered the risk of AF-related outcomes, including hospitalization, compared with usual care.^181^ The MONITOR AF study showed that implantable loop recorder–guided monitoring led to earlier intervention, improved rhythm control, and reduced AF and HF hospitalizations.^182^

### Cost of illness and cost-effectiveness in recent-onset atrial fibrillation

Estimates of the medical costs for health care in AF patients vary substantially.^183–185^ In a systematic review, stroke and HF were responsible for a large share of the total costs; therefore, proactive management of co-morbidities in AF can improve health and mitigate healthcare costs.^186,187^ Early or timely detection of AF in some subgroups of at-risk patients may allow intervention, possibly preventing the progression of the disease and some major healthcare costs, especially from stroke.^187,188^ This has been shown to be cost-effective with an incremental cost-effectiveness ratio per quality-adjusted life year gained below a €50 000 threshold.^189–192^ Early rhythm-control therapy has been associated with a lower risk of stroke and cardiovascular outcomes than usual care among AF patients with cardiovascular conditions.^1,180,193^

### Anxiety/quality of life

Anxiety is prevalent in approximately one-third of AF patients, with a higher prevalence in women.^194–196^ Anxiety and reduced QoL can be consequences of being diagnosed with AF and/or related to symptoms and their severity, however a bi-directional relationship may exist with anxiety triggering AF.^197^ Stress (acute and chronic) induces autonomic dysregulation, triggers inflammatory responses (endocrine, immune, neuronal, and vascular), and promotes detrimental health behaviours (impaired sleep, poor diet, physical inactivity, smoking, alcohol consumption), which may result in AF.^198,199^ Symptomatic AF patients report greater prevalence and severity of anxiety,^200^ due to unpredictable onset, frequency, and severity of symptoms, which limit daily activities and negatively impact QoL.^194,195,201^ Fear of AF complications, particularly stroke, OAC-associated bleeding,^202^ medication concerns, and uncertainty about the future, increases anxiety.^203^ Rhythm control strategies, particularly catheter ablation, are associated with a reduction in anxiety^204^ and improvements in QoL^86,205^ mainly related to symptomatic relief, but there is currently no evidence of benefit of earlier treatment improving these outcomes.

## Potential disadvantages of early intervention for recent-onset atrial fibrillation

When patients with symptomatic or sustained forms of AF present, management is clear and might lead to the reduction of symptoms previously not attributed to AF.

For recent-onset AF, characterized by short, sporadic, asymptomatic AF episodes, management is more of a challenge as treatment strategies are less clear. Establishing the diagnosis provides opportunities for therapy and risk reduction but also creates anxiety and may lead to additional care, thus creating a treatment burden, and a need for information. Many will be subjected to investigations, largely safe but with occasional complications and associated with costs.

The least harmful management, the need to modify lifestyle, may be greeted by distress. When therapy is recommended purely to prevent the emergence or progression of AF, for example, an anti-arrhythmic drug, the therapeutic efficacy of the treatment should be carefully considered in the light of unwanted adverse effects of the drug. The possibility of lifelong therapy is expensive for both patients and healthcare providers.

Should an invasive therapy be contemplated, the short-term risk of the intervention must be weighed against any long-term therapeutic gain. The lifelong efficacy of catheter ablation for rhythm control or left atrial appendage closure for thromboprophylaxis is not yet known.

## Research opportunities to address knowledge gaps

More data are needed to guide the best management of recent-onset AF, bearing AF burden and AF progression patterns in mind. Long-term studies using large national databases linked to electronic medical records and to rhythm monitoring devices offer excellent opportunities for better defining recent-onset AF and designing trials for its treatment. Shorter-term studies focusing on reducing AF burden to slow AF progression could be designed in both randomized clinical trials and observational cohort studies. Supplementary data online, *Table S1* details a list of future studies that are warranted. When undertaking clinical studies of recent-onset AF, it should be kept in mind that enrolment of low-risk populations with inadequate sample size and too short follow-up duration carries a substantial risk of false negative findings with regard to identifying AF progression and AF-related complications.

While sex-related differences are well documented in broader AF populations, their relevance in the context of recent-onset AF remains insufficiently explored. Future studies should assess whether the clinical presentation, progression, or treatment response differs by sex in patients with recent-onset AF to inform about more individualized care strategies.

## Summary and conclusions

Early management of recent-onset AF should be considered since AF detection at an early stage is increasing. Prior studies have mainly focused on the prevention of ischaemic stroke in AF patients, but other important outcomes, including HF and cognitive decline, are emerging and should be a focus of future trials of recent-onset AF.

Effective treatment of underlying cardiovascular co-morbidities and risk factor management is not only safe but may also slow the progression of AF and reduce the risk of future cardiovascular events. Anticoagulation might be warranted, but the potential benefit needs to be carefully balanced against the risk of bleeding if the calculated risk of stroke is low. Early rhythm control, alongside anticoagulation, reduces symptoms and the burden of AF. As technologies continue to advance, this approach is likely to gain popularity and may contribute to improved long-term outcomes. However, medical interventions to resolve AF are associated with potential adverse complications, and the rush to implement them early in the course of the disease warrants caution. Studies are ongoing to determine if rhythm control early after recent-onset AF may reduce the need for thromboprophylaxis. Finally, the relative benefits of rhythm control vs addressing underlying atrial myopathy and other co-morbidities also require further elucidation. A cautious but progressive course is recommended.

## Supplementary Material

ehaf478_Supplementary_Data

## Declarations

### Data Availability

No data were generated or analysed for or in support of this paper.
