# Supplementary material for: Recent-onset atrial fibrillation: challenges and opportunities
Source: Eur Heart J. 2025 Aug 28;47(2):170–87. doi: 10.1093/eurheartj/ehaf478 (PMC12777708; doi:10.1093/eurheartj/ehaf478)
Supplement: ehaf478_Supplementary_Data [file ehaf478_supplementary_data.zip › Abbreviations.docx]

# Abbreviations (trials, registries and scores)

**ACTIVE-AF:** An Exercise and Physical Activity Program in Patients With Atrial Fibrillation.

**ARCADIA:** AtRial Cardiopathy and Antithrombotic Drugs In Prevention After Cryptogenic Stroke. (NCT03192215).

**ARIC AF:** Atherosclerosis Risk in Communities – Atrial Fibrillation.

**ARTESiA:** Apixaban for Stroke Prevention in Subclinical Atrial Fibrillation.

**ASSERT:** Asymptomatic Atrial Fibrillation and Stroke Evaluation in Pacemaker Patients and the Atrial Fibrillation Reduction Atrial Pacing Trial.

**ASSERT II:** Prevalence of Sub-Clinical Atrial Fibrillation Using an Implantable Cardiac Monitor. (NCT01694394).

**ATHENA:** A Trial With Dronedarone to Prevent Hospitalization or Death in Patients With Atrial Fibrillation. (NCT00174785).

**BRAIN-AF:** Blinded Randomized Trial of Anticoagulation to Prevent Ischemic Stroke and Neurocognitive Impairment in AF (NCT02387229).

**C_2_HEST**: Coronary artery disease or chronic obstructive pulmonary disease 1 point each; hypertension 1 point; elderly [age ≥75 years] 2 points; systolic heart failure, 2 points; thyroid disease; hyperthyroidism, 1 point.

**CABA-HFpEF:** CAtheter-Based Ablation of Atrial Fibrillation Compared to Conventional Treatment in Patients With Heart Failure With Preserved Ejection Fraction. (NCT05508256).

**CABANA:** Catheter Ablation vs Anti-arrhythmic Drug Therapy for Atrial Fibrillation Trial. (NCT00911508).

**CHA_2_DS_2_-VASc:** Cardiac failure or dysfunction, Hypertension, Age >=75 [Doubled], Diabetes, Stroke [Doubled]-Vascular disease, Age 65-74, and Sex category [Female].

**CHADS_2_:** Congestive heart failure, Hypertension, Age ≥75 years, and diabetes mellitus, each given 1 point; and a past history of transient ischemic attack or stroke: 2 points.

**CHARGE AF:** age, ethnicity, height, weight, systolic blood pressure (SBP), diastolic blood pressure (DBP), current smoking, antihypertensive medication use, diabetes mellitus (DM), heart failure and myocardial infarction (MI).

**EAST-AFNET 11:** not registered.

**EAST-AFNET 4:** Early Treatment of Atrial Fibrillation for Stroke Prevention Trial. (NCT01288352).

**FIND-AF:** Future Innovations in Novel Detection for Atrial Fibrillation. (NCT05898165).

**GARFIELD-AF:** Global Anticoagulant Registry in the Field - Atrial Fibrillation.

**HARMS2-AF:** Hypertension, sleep apnoea, male sex, age, obesity (BMI>30kg/m^2^), alcohol and smoking.

**HATCH:** Hypertension:1 point, Age >75 years old: 1 point, transient ischemic attack or stroke: 2 points, chronic obstructive pulmonary disease: 1 point, and heart failure: 2 points.

**I-STOP-AFib:** Individualized Studies of Triggers of paroxysmal Atrial Fibrillation. (NCT03323099).

**LOOP:** Atrial Fibrillation Detected by Continuous ECG Monitoring. (NCT02036450).

**MAFA II:** Mobile Atrial Fibrillation Application II. (ChiCTR-OOC-17014138).

**NOAH-AFNET 6:** Non-vitamin K Antagonist Oral Anticoagulants in Patients with Atrial High Rate Episodes. (NCT02618577).

**NOR-SCREEN:** NORwegian Atrial Fibrillation Self-SCREENing. (NCT05914883).

**RACE 3:** Routine Versus Aggressive Upstream Rhythm Control for Prevention of Early Atrial Fibrillation in Heart Failure. (NCT00877643).

**RECORD AF:** REgistry on Cardiac rhythm disORDers assessing the control of Atrial Fibrillation.

**REVERSE-AF:** PREVEntion and regReSsive Effect of weight-loss and risk factor modification on Atrial Fibrillation. (ACTRN12614001123639).

**SAFER:** Screening for atrial fibrillation with ECG to reduce stroke. (ISRCTN16939438).

**STROKESTOP:** Systematic ECG Screening for Atrial Fibrillation Among 75 Year Old Subjects in the Region of Stockholm and Halland, Sweden. (NCT01593553).

**STROKESTOP II:** Systematic NT-proBNP and ECG Screening for Atrial Fibrillation Among 75 Year Old Subjects in the Region of Stockholm, Sweden. (NCT02743416).
