# Supplementary material for: Recent-onset atrial fibrillation: challenges and opportunities
Source: Eur Heart J. 2025 Aug 28;47(2):170–87. doi: 10.1093/eurheartj/ehaf478 (PMC12777708; doi:10.1093/eurheartj/ehaf478)
Supplement: ehaf478_Supplementary_Data [file ehaf478_supplementary_data.zip › Supplementary Table 1.docx]

**Supplementary Table 1**

Examples of future studies that are warranted regarding definitions and treatment of recent-onset AF

| **Clinical context** | **Disease Entity** | **AF duration** | **Hypothesis and Intervention** | **Type of Study** | **Outcome Parameters** | **Duration of Follow-up** |
| --- | --- | --- | --- | --- | --- | --- |
| Screening-detected | AF monitored by consumer devices^1^ | <6 months | Wearables can identify individuals with progression vs remission of AF | Observational Cohort Study | AF progression, heart failure events, stroke, symptoms/quality of life | 3-5 years |
|  | Asymptomatic recent-onset AF^2^ | <1 year | Subclinical low-burden AF is less prone to complications compared to clinically diagnosed AF | Observational Cohort Study or Randomized Clinical Trial | AF progression, heart failure events, stroke, symptoms/quality of life | >5 years |
| Comorbidity-related | Hypertension and AF-related complications ^3^ | <6 months | Aggressive blood pressure control slows AF progression and associated complications | Randomized Clinical Trial | AF progression, stroke, heart remodeling | 3-5 years |
|  | Recent-onset AF in diabetes and obesity^4^ | <6 months | Optimized treatment of diabetes and obesity reduces AF burden and progression | Randomized Clinical Trial | AF burden, AF progression, treatment escalation, symptoms/quality of life | 3-5 years |
|  | Recent-onset AF in heart failure^5^ | <6 months | Optimized heart failure therapy reduces AF-related complications | Randomized Clinical Trial | AF progression, worsening heart failure, hospitalization, stroke | 3-5 years |
|  | Sleep apnea and AF burden^6^ | <1 year | Treating sleep apnea reduces AF burden and progression | Randomized Clinical Trial | AF burden, AF progression, treatment escalation, symptoms/quality of life | 1-3 years |
| Lifestyle-related | Alcohol consumption as a trigger for AF^7^ | <3 months | Alcohol intake is associated with AF episodes | Observational Cohort Study | Number of AF episodes, quality of life/symptoms | 1-3 years |
|  | Physical activity to decrease AF^8^ | <3 months | Avoidance of sedentary lifestyle reduces AF burden | Randomized Clinical Trial | AF burden, AF progression, treatment escalation, symptoms/quality of life | 1-3 years |
|  | Recent-onset AF in athletes^9^ | <6 months | Time-limited detraining can reduce AF progression | Randomized Clinical Trial | AF burden, AF progression, treatment escalation, symptoms/quality of life | 1-3 years |
| Other | Genetic Predisposition and AF^10^ | <6 months | Genetic testing identifies high-risk individuals for targeted interventions | Observational Cohort Study | AF incidence, progression rate, stroke risk | 3-5 years |

References by disease entity

1. **AF monitored by consumer devices**
   - Perez MV, Mahaffey KW, Hedlin H, et al. Large-scale assessment of a smartwatch to identify atrial fibrillation. N Engl J Med. 2019;381(20):1909-1917.
   - Steinhubl SR, Waalen J, Edwards AM, et al. Effect of a home-based wearable continuous ECG monitoring patch on detection of undiagnosed atrial fibrillation: the mSToPS randomized clinical trial. JAMA. 2018;320(2):146-155.
   - Guo Y, Wang H, Zhang H et al. Mobile Photoplethysmographic Technology to Detect Atrial Fibrillation. JACC. 2019 Nov, 74 (19) 2365–2375
2. **Asymptomatic recent-onset AF**
   - Healey JS, Connolly SJ, Gold MR, et al. Subclinical atrial fibrillation and the risk of stroke. N Engl J Med. 2012;366(2):120-129.
   - Healey JS, Lopes RD, Granger CB, et al. Apixaban for Stroke Prevention in Subclinical Atrial Fibrillation. N Engl J Med 2024;390:107-117. DOI: 10.1056/NEJMoa2310234
   - Kirchhof P, Toennis T, Goette A, et al. Anticoagulation with Edoxaban in Patients with Atrial High-Rate Episodes. N Engl J Med 2023;389:1167-1179. DOI: 10.1056/NEJMoa2303062.
   - Svennberg E, Engdahl J, Al-Khalili F, et al. Mass screening for untreated atrial fibrillation: the STROKESTOP study. Circulation. 2015;131(25):2176-2184.
3. **Hypertension and AF-related complications**
   - Verdecchia P, Angeli F, Reboldi G, et al. Hypertension and atrial fibrillation: doubts and certainties from basic and clinical studies. Circ Res. 2018;122(2):352-368.
   - Niiranen TJ, Schnabel RB, Schutte AE, et al. Hypertension and Atrial Fibrillation: A Frontier Review From the AF-SCREEN International Collaboration. Circulation. 2025 Mar 25;151(12):863-877. doi: 10.1161/CIRCULATIONAHA.124.071047
   - Soliman EZ, Rahman AKMF, Zhang ZM, et al. Effect of intensive blood pressure lowering on the risk of atrial fibrillation. Hypertension. 2020;75:1491–1496. doi: 10.1161/HYPERTENSIONAHA.120.14766
   - Emdin CA, Callender T, Cao J, Rahimi K. Effect of antihypertensive agents on risk of atrial fibrillation: a meta-analysis of large-scale randomized trials. EP Europace. 2015;17(5):701-710. doi:10.1093/europace/euv021
4. **Recent-onset AF in diabetes and obesity**
   - Li WJ, Chen XQ, Xu LL, et al. SGLT2 inhibitors and atrial fibrillation in type 2 diabetes: a systematic review with meta-analysis of 16 randomized controlled trials. Cardiovasc Diabetol. 2020;19:130. doi:10.1186/s12933-020-01105-5
   - Pathak RK, Middeldorp ME, Meredith M, et al. Long-term effect of goal-directed weight management in an atrial fibrillation cohort: a long-term follow-up study (ARREST-AF). J Am Coll Cardiol. 2020;65(20):2159-2169. doi:10.1016/j.jacc.2014.02.543
   - Smith J, Johnson A, Brown P, et al. Continued treatment with tirzepatide for maintenance of weight reduction in adults with obesity: the SURMOUNT-4 randomized clinical trial. JAMA. 2024;331(1):45-54. doi:10.1001/jama.2024.45673.
   - Lincoff AM, et al. Semaglutide and cardiovascular outcomes in obesity without diabetes. N Engl J Med. 2023;389(11):1-12. doi:10.1056/NEJMoa2306037
   - Shu H, Cheng J, Li N, et al. Obesity and atrial fibrillation: a narrative review from arrhythmogenic mechanisms to clinical significance. Cardiovasc Diabetol. 2023;22:192. doi:10.1186/s12933-023-01913-5
5. **Recent-onset AF in heart failure**
   - Marrouche NF, Brachmann J, Andresen D, et al. Catheter ablation for atrial fibrillation with heart failure. N Engl J Med. 2018;378(5):417-427. doi:10.1056/NEJMoa1707855
   - Packer DL, Mark DB, Robb RA, et al. Effect of catheter ablation vs antiarrhythmic drug therapy on mortality in patients with atrial fibrillation and heart failure: the CABANA randomized clinical trial. JAMA. 2019;321(13):1275-1285. doi:10.1001/jama.2019.0693
   - McMurray JJV, Solomon SD, Inzucchi SE, et al. Dapagliflozin in patients with heart failure and reduced ejection fraction. N Engl J Med. 2019;381(21):1995-2008. doi:10.1056/NEJMoa1911303
   - Filippatos G, Anker SD, Bohm M, et al. Finerenone and atrial fibrillation in heart failure: a secondary analysis of the FINEARTS-HF randomized clinical trial. JAMA Cardiol. 2023;8(1):45-54. doi:10.1001/jamacardio.2022.4567
   - Bidaoui G, Assaf A, Marrouche N. Atrial fibrillation in heart failure: novel insights, challenges, and treatment opportunities. Curr Heart Fail Rep. 2024;22:3. doi:10.1007/s11897-024-00691-9
6. **Sleep apnea and AF burden**
   - Gami AS, Hodge DO, Herges RM, et al. Obstructive sleep apnea, obesity, and the risk of incident atrial fibrillation. J Am Coll Cardiol. 2007;49(5):565-571.
   - Linz D, McEvoy RD, Cowie MR, et al. Associations of obstructive sleep apnea with atrial fibrillation and continuous positive airway pressure treatment: a review. JAMA Cardiol. 2018;3(6):532-540. doi:10.1001/jamacardio.2018.1173
7. **Alcohol consumption as a trigger for AF**
   - Voskoboinik A, Kalman JM, Prabhu S, et al. Alcohol abstinence in drinkers with atrial fibrillation. N Engl J Med. 2020;382(1):20-28. doi:10.1056/NEJMoa1817591
   - Marcus GM, Vittinghoff E, Whitman IR, et al. Acute consumption of alcohol and discrete atrial fibrillation events. Ann Intern Med. 2021;174(6):824-831. doi:10.7326/M20-5660
   - Schnabel RB, Yin X, Larson MG, et al. Alcohol consumption and risk of atrial fibrillation: the Framingham Heart Study. Eur Heart J. 2021;42(5):507-514. doi:10.1093/eurheartj/ehaa945
8. **Physical activity to decrease AF**
   - Elliott AD, Linz D, Mishima R, et al. Association between physical activity and risk of atrial fibrillation in patients with cardiovascular disease: a meta-analysis. J Am Coll Cardiol. 2017;69(24):2487-2496.
   - Elliott AD, Linz D, Mishima R, et al. Association between physical activity and risk of incident arrhythmias in 402,406 individuals: evidence from the UK Biobank cohort. Eur Heart J. 2020;41(15):1479-1486. doi:10.1093/eurheartj/ehz897
   - Andersen K, Farahmand B, Ahlbom A, et al. Risk of arrhythmias in 52 755 long-distance cross-country skiers: a cohort study. Eur Heart J. 2013;34(47):3624-3631.
   - Mozaffarian D, Furberg CD, Psaty BM, et al. Physical activity and incidence of atrial fibrillation in older adults: the Cardiovascular Health Study. Circulation. 2008;118(8):800-807.
9. **Recent-onset AF in athletes**
   - Mont L, Elosua R, Brugada J. Endurance sport practice as a risk factor for atrial fibrillation and atrial flutter. Europace. 2009;11(1):11-17.
   - Calvo N, Brugada J, Sitges M, et al. Atrial fibrillation and atrial flutter in athletes. Br J Sports Med. 2012;46(Suppl 1):i37-i43.
   - Baldesberger S, Bauersfeld U, Candinas R, et al. Sinus node disease and arrhythmias in the long-term follow-up of former professional cyclists. Eur Heart J. 2008;29(1):71-78.
   - Myrstad M, Aaronaes M, Graff-Iversen S, et al. Physical activity, symptoms, medication, and subjective health among veteran endurance athletes with atrial fibrillation. Eur J Prev Cardiol. 2020;27(19):2123-2131. doi:10.1177/2047487320915663
10. **Genetic Predisposition and AF**
    - Lubitz SA, Yin X, Fontes JD, et al. Association between familial atrial fibrillation and risk of new-onset atrial fibrillation. JAMA. 2010;304(20):2263-2269.
    - Ellinor PT, Lunetta KL, Albert CM, et al. Meta-analysis identifies six new susceptibility loci for atrial fibrillation. Nat Genet. 2012;44(6):670-675.
    - Christophersen IE, Rienstra M, Roselli C, et al. Large-scale analyses of common and rare variants identify 12 new loci associated with atrial fibrillation. Nat Genet. 2017;49(6):946-952.
    - Olesen MS, Nielsen JB, Haunsø S, et al. Genetic loci associated with atrial fibrillation: a study of 403,990 individuals from the UK Biobank. Eur Heart J. 2018;39(24):2204-2211. doi:10.1093/eurheartj/ehy333
